# Silent Signals: Correlating Plant Bioelectric Activity with Human Emotional States via Wearable Sensing

**DOI:** 10.3390/biomimetics11040236

**Published:** 2026-04-02

**Authors:** Peter A. Gloor

**Affiliations:** 1 MIT System Design Management, Massachusetts Institute of Technology, Cambridge, MA 02139, USA; pgloor@mit.edu; 2 Cologne Institute of Information Science, University of Cologne, 50923 Cologne, Germany; 3 Galaxyadvisors AG, 5000 Aarau, Switzerland

**Keywords:** plant bioelectrics, bio-hybrid sensing, emotion sensing, wearable computing, biosignal processing, human–plant interaction, ambient intelligence, fixed-effects model

## Abstract

We present a bio-hybrid sensing system that uses a living plant (*Tradescantia pallida*) as an ambient biosensor for human stress states as a single-participant proof-of-concept study. An AD8232 biosignal amplifier captures plant bioelectric activity, while a Happimeter smartwatch simultaneously measures the wearer’s mood via machine learning on wrist-worn sensor data. Over 129 paired observations across eleven days in a naturalistic desk-work setting, a within-day fixed-effects analysis reveals robust stress–plant coupling: seven correlations survive Benjamini–Hochberg false discovery rate correction (*q* = 0.05), with two also surviving Bonferroni correction. The strongest results are stress_rolling vs. plant mean (r = +0.36, *p* = 3.3 × 10^−5^) and RMS (r = +0.34, *p* = 7.8 × 10^−5^). An incidental electrode reattachment mid-experiment created a natural control: mean/RMS correlation signs flipped with electrode polarity, while the coefficient of variation remained consistently negative across both configurations (r = −0.32, *p* = 2.6 × 10^−4^). This electrode-invariant finding—higher stress associated with lower relative signal variability—provides the strongest evidence for genuine bio-hybrid sensing. The results position living plants as bio-inspired ambient sensing elements for workplace wellbeing monitoring.

## 1. Introduction

The emerging field of plant electrophysiology has demonstrated that plants exhibit complex electrical signaling in response to environmental stimuli, including light, touch, sound, and chemical exposure [1,2,3,4,5,6,7]. Our recent work has shown that machine learning classifiers can distinguish plant bioelectric responses to environmental stimuli with 85% accuracy and human emotional states with 73% accuracy using ResNet18 CNNs applied to mel-spectrograms of *Tradescantia pallida* signals [8]. In a separate five-year investigation, we demonstrated that plants generate distinct bioelectric signatures correlating with human proximity and movement, achieving 63% classification accuracy with Random Forest and identifying performer-specific signatures in plant recordings [9].

Parallel advances in wearable emotion recognition have produced systems capable of inferring emotional states from physiological signals [10,11]. The Happimeter platform, developed at MIT’s Center for Collective Intelligence, uses smartwatch sensors combined with machine learning to continuously predict mood dimensions including happiness, stress, and depression. Roessler and Gloor [12] demonstrated that wearing the Happimeter and receiving mood feedback over three months significantly increased self-reported happiness in a controlled experiment with 22 participants.

Despite this convergence of plant electrophysiology and wearable emotion sensing, a critical gap remains: no prior study has investigated continuous, real-time correlations between plant bioelectric signals and human mood in a naturalistic setting. Existing plant-based emotion work—including our own classification studies [8,13,14] and Smolin et al.’s stress prediction work [15]—relies on discrete, experimenter-controlled stimulus events and short recording windows, precluding observation of the slow co-variation between sustained mood states and ongoing plant electrical activity. Equally, wearable stress detection systems [10,16] operate without any plant sensing component. The present study fills this gap by treating the plant as a continuous ambient sensor correlated in real time against wearable mood ground truth across eleven naturalistic working days.

In this paper, we bridge these two research streams: continuously recording plant bioelectric signals while simultaneously collecting mood predictions from the Happimeter smartwatch. Unlike our previous classification-based approaches [8,9], which used discrete stimulus categories, we investigate whether continuous, real-time correlations exist between plant bioelectric features and human mood dimensions during naturalistic desk work.

We report results from 129 paired observations over eleven days, employing within-day fixed-effects analysis to control for between-day confounds. An incidental electrode reattachment mid-experiment created a natural control that separates electrode-dependent from electrode-invariant associations. Seven stress–plant correlations survive Benjamini–Hochberg false discovery rate correction [4], with the electrode-invariant coefficient of variation providing the most robust evidence for genuine bio-hybrid sensing.

## 2. Related Work

### 2.1. Plant Bioelectric Sensing

Plants generate electrical signals through ion channel activity, producing action potentials and variation potentials in response to stimuli [17]. Fromm and Lautner [18] demonstrated that these signals propagate through the phloem and can be measured non-invasively using surface electrodes [5]. Chatterjee et al. [6,7] showed that machine learning classifiers can distinguish plant responses to different environmental stimuli using curve-fitting features. Our prior work established the foundation for the present study: in Gloor [8], we achieved 85.4% accuracy for environmental stimulus classification and 73% for emotion classification using *Tradescantia pallida* signals, while a five-year investigation [9] demonstrated performer-specific plant bioelectric signatures at 63% classification accuracy across multiple plant species.

### 2.2. Wearable Emotion Recognition

The Happimeter system [12] predicts individual emotions from a Google Pixel Watch smartwatch (Google LLC, Mountain View, CA, USA) running WearOS sensor data (acceleration, heartbeat, and activity level). In a three-month deployment with 22 bank employees, participants receiving mood feedback showed significantly increased happiness compared to controls. The system employs an XGBoost classifier predicting happiness, stress, and depression on a 0–2 scale at intervals of 10–20 min.

### 2.3. Human–Plant Interaction

Research at the intersection of human–plant interaction has explored psychological benefits of plant proximity [19,20,21] and plant-mediated communication interfaces [11]. The concept of plants as biosensors has been investigated for environmental monitoring [22], but its extension to continuous real-time human emotion correlation remains unexplored. Our approach treats the plant as a continuous ambient sensor correlated with wearable ground truth, rather than using discrete stimulus–response classification paradigms.

Table 1 resumes the state of the art in plant bioelectric sensing, wearable emotion recognition and human–plant interaction.

## 3. System Architecture

**Figure 1:**
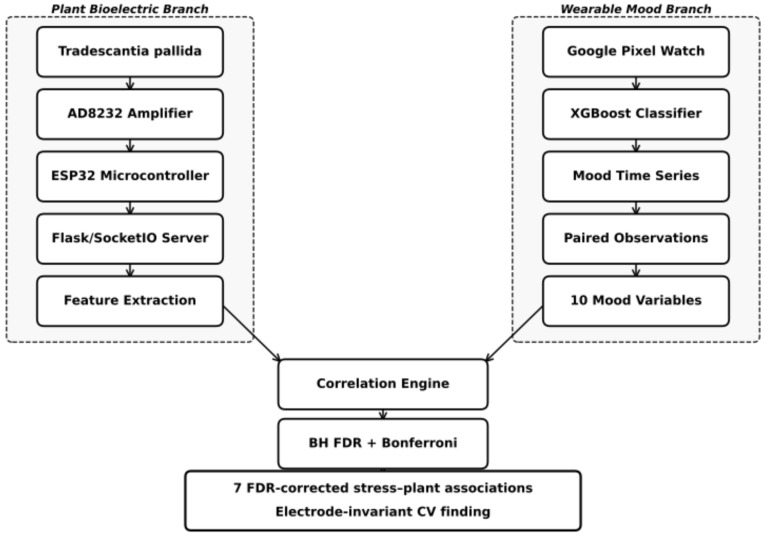
System architecture of the bio-hybrid sensing pipeline. (**Left**): plant bioelectric acquisition branch. (**Right**): Happimeter wearable mood-tracking branch. (**Centre**): experimental context and the Biolingo Wellbeing Correlator analysis engine.

Figure 1 provides an overview of the complete bio-hybrid sensing pipeline, illustrating the two parallel data acquisition branches—plant bioelectric and wearable mood tracking—and their convergence in the Biolingo Wellbeing Correlator for fixed-effects correlation analysis.

### 3.1. Plant Bioelectric Sensor (Biolingo)

**Figure 2:**
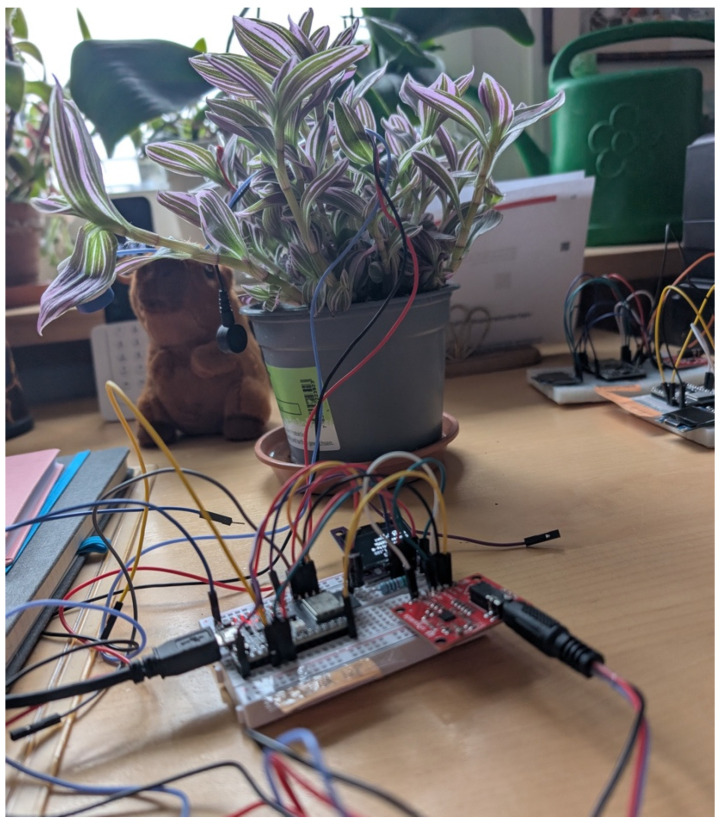
AD8232 biosignal amplifier on breadboard connected to ESP32 microcontroller, with electrode leads attached to the *Tradescantia pallida* plant.

The plant sensing subsystem consists of an AD8232 single-lead biosignal amplifier connected to a *Tradescantia pallida* (Purple Heart) via silver-chloride adhesive electrodes attached to two leaves, with a reference electrode on the stem (Figure 2). The AD8232 provides instrumentation-grade amplification with a built-in high-pass filter at approximately 0.5 Hz and gain of approximately 100×, operating at a ~120 Hz sampling rate for continuous long-duration recording. The amplified signal is digitized by an ESP32 microcontroller's 12-bit ADC and transmitted via WebSocket to a Python (v3.11.13) Flask/SocketIO (Flask v3.1.1, Flask-SocketIO v5.4) server.

The AC-coupled differential measurement means that the sign of the measured voltage depends on the orientation and placement of the two leaf electrodes relative to each other and the reference. This has important implications for interpretation: the absolute direction of mean voltage changes is electrode-configuration-dependent, while normalized metrics, such as the coefficient of variation, are invariant to electrode polarity. Care must be taken to electrically isolate the AD8232 sensing circuit from other devices sharing the ESP32’s power rail, as ground loops through USB-connected peripherals can saturate the ADC at the supply voltage.

### 3.2. Mood Tracking via Happimeter

The Happimeter Google Pixel Watch application [12] collects 45 s bursts of sensor data (accelerometer, microphone, and heart rate) at scheduled intervals of 10–20 min. The system predicts three mood dimensions on a scale of 0–2: happiness, stress, and depression. Due to WearOS power management constraints, some scheduled readings were missed, producing gaps in the mood time series.

### 3.3. Correlation Engine

**Figure 3:**
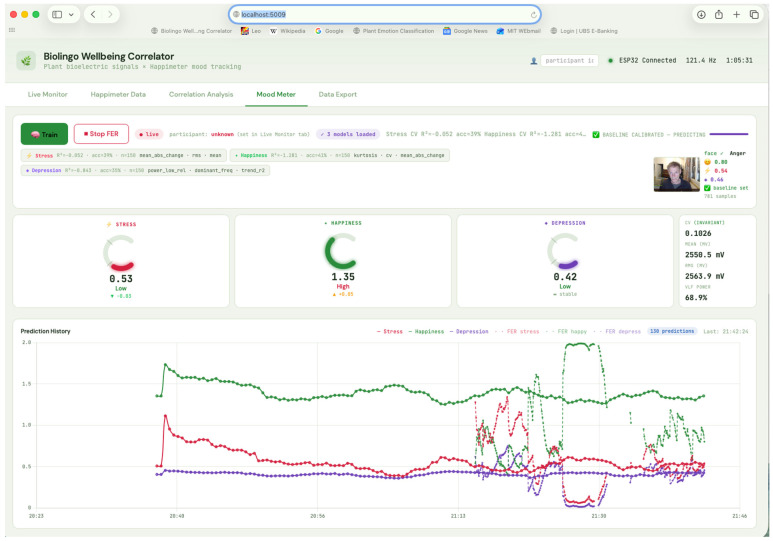
The Biolingo Wellbeing Correlator web interface showing real-time plant bioelectric signal monitoring and correlation analysis.

The Biolingo Wellbeing Correlator, a Python Flask (v3.1.1)/SocketIO (v5.1.1) web application (Figure 3), extracts 20 features from the 5 min window of raw plant voltage preceding each Happimeter reading: statistical (mean, std, range, IQR, CV, and RMS), temporal (trend slope/R^2^, direction change rate, jitter, and mean absolute change), frequency (total power, dominant frequency, and relative power in 5 sub-bands), and shape (skewness, kurtosis, and peak count/rate). Ten mood variables (3 raw, 2 composites, and 5 rolling averages) are each correlated with 20 plant features, yielding 200 tests per analysis level.

We distinguish three analysis levels throughout: Level 1 (pooled standard correlations; n = 129), Level 2 (within-day fixed effects via day-demeaning; n = 128), and Level 3 (within-day fixed effects with additional circadian detrending, removing a linear intra-day time trend from both plant and mood variables within each day before correlation). Level 2 removes all between-day confounds by construction, and Level 3 additionally controls for smooth within-day temporal variation in the plant signal, which prior work has shown accounts for approximately 14% of RMS signal variance due to circadian rhythms.

## 4. Experimental Setup

Data was collected over eleven days (19 February–1 March 2026) in a home office in Aarau, Switzerland. A single participant (male, age 64) wore a Google Pixel Watch running the Happimeter application (v.2.7) while working at a desk approximately 40–60 cm from the instrumented *Tradescantia pallida*. The ESP32 sensor streamed plant voltage continuously at ~120 Hz.

On 25 February, a USB power cable for an unrelated solenoid device was connected to the shared power circuit, creating a ground loop that saturated the AD8232 at its supply voltage (3.3 V). After identifying and removing the contaminated observations, the electrodes were reattached to different leaves of the same plant. This created two electrode configuration eras: Era A (19–24 February; original placement; 81 observations across 6 days) and Era B (25 February–1 March; reattached placement; 48 observations across 5 days). The two eras produced plant signals with substantially different baselines (Era A mean: 24–188 mV; Era B mean: 30–1827 mV). This incidental electrode change provided a natural control for separating electrode-dependent from electrode-invariant associations.

The 25–40× increase in mean plant signal amplitude between eras (Era A mean: 24–188 mV; Era B mean: 30–1827 mV) reflects the sensitivity of the AC-coupled differential measurement to electrode placement geometry. Repositioning the two leaf electrodes to different leaves altered the inter-electrode distance, the thickness and vascular density of the intervening leaf tissue, and the proximity of each electrode to the midrib—all of which influence the differential voltage captured by the AD8232 instrumentation amplifier. This is consistent with established plant electrophysiology practice, in which signal amplitude varies substantially with electrode placement even on the same plant [23,25]. Critically, the within-day fixed-effects analysis removes between-day baseline differences by construction, and the electrode-invariant CV metric is unaffected by absolute signal magnitude.

In total, 129 observations met the matching criterion (≥10 plant samples in the preceding 5 min window) (Table 2). Ten days had ≥2 observations and were included in the fixed-effects analysis (128 observations): February 20 (19), 21 (16), 22 (14), 23 (19), 24 (12), 25 (7), 26 (17), 27 (12), and 28 (2) and March 1 (10). One observation from February 19 was excluded as the sole entry for that day.

Although eleven days constitutes a modest recording period, the resulting 128 paired observations provide substantial statistical power for the correlation analyses performed. For the observed effect sizes (FE r ≈ 0.32–0.36), a post hoc power analysis using the formula for Pearson correlation [26] yields power >0.99 at α = 0.05 and n = 128, far exceeding the conventional threshold of 0.80. Furthermore, the within-day fixed-effects design requires only that each day contributes ≥2 observations for inclusion, which all ten analysis days satisfy; the number of days determines the number of independent confound-control units rather than raw statistical power.

## 5. Results

### 5.1. Within-Day Fixed-Effects Analysis

Given the multiple electrode configurations in our dataset, standard pooled correlations conflate between-day differences in electrode placement, weather, sleep quality, and other confounds with within-day mood–plant co-variation. We therefore present the within-day fixed-effects analysis [26,27] as our primary result.

For each variable, we subtract the daily mean (day-demeaning), isolating only within-day variation. This removes all time-invariant daily confounds by construction, including electrode configuration effects on the signal baseline. We analyzed 128 observations from ten days, with ≥2 observations each.

The fixed-effects results (Table 3) reveal 19 significant correlations (*p* < 0.05) out of 200 tests. Seven survive Benjamini–Hochberg false discovery rate correction at *q* = 0.05, all involving stress-related mood dimensions. The two strongest—stress_rolling vs. mean (r = +0.358, *p* = 3.3 × 10^−5^) and stress_rolling vs. RMS (r = +0.342, *p* = 7.8 × 10^−5^)—also survive Bonferroni correction (α/200 = 2.5 × 10^−4^). The seven survivors span three mood variables (stress_rolling, raw stress, and negative_affect_rolling) and two plant features (mean, RMS, and CV), demonstrating that the effect generalizes across the broader stress construct rather than depending on a single operationalization.

As the most conservative robustness check, we applied circadian detrending within each day prior to correlation (Level 3), removing the linear intra-day time trend from both signals. Of the 19 Level 2 significant correlations, 9 survive at Level 3 (*p* < 0.05) out of 200 tests, compared to the ~10 expected by chance. The composition of survivors shifts: stress_rolling × mean and stress_rolling × RMS—the two Bonferroni-corrected Level 2 survivors—do not survive circadian detrending, indicating that part of the mean/RMS coupling is shared with the plant’s intra-day drift. This is expected, since the AC-coupled mean voltage carries a circadian component that partially co-varies with the within-day stress trajectory. Crucially, the electrode-invariant metric does survive: stress_rolling × CV remains significant at Level 3 (r = −0.211, *p* = 0.017), confirming that the suppression of relative signal variability under stress is robust to both electrode configuration and circadian detrending. The Level 3 survivors reveal additional stress-related associations in the frequency and temporal domains: stress_rolling × power_very_low_rel (r = +0.346, *p* < 0.001), stress_rolling × mean_abs_change (r = −0.229, *p* = 0.010), stress_rolling × jitter (r = −0.201, *p* = 0.023), and stress_rolling × trend_r^2^ (r = −0.177, *p* = 0.045). That stress_rolling associations dominate across all three analysis levels—spanning statistical, frequency, and temporal plant features—argues for a genuine and multi-faceted stress–plant coupling rather than a single shared confound.

### 5.2. Electrode Configuration and Polarity Invariance

**Figure 4:**
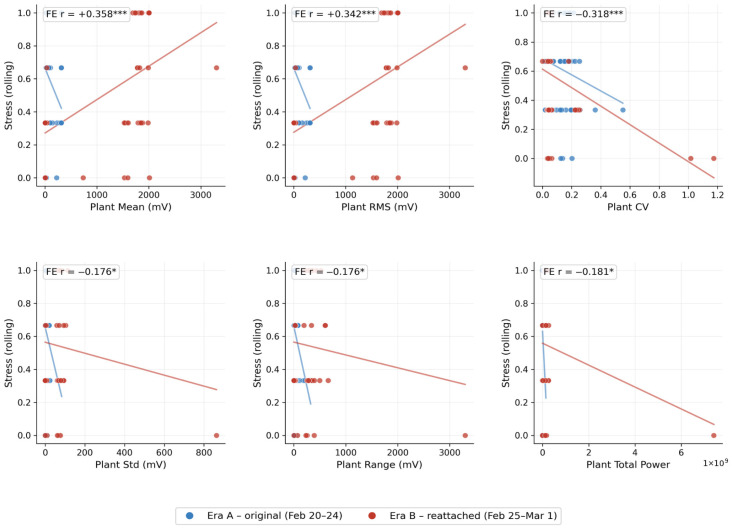
Stress–plant scatter plots by electrode configuration (n = 129). Top row: plant mean and RMS show opposite regression slopes between Era A (blue; Feb 20–24) and Era B (red; Feb 25–Mar 1), with plant CV consistently negative. Bottom row: variability metrics (std, range, and total power) are negative in both eras. FE r values are from the day-demeaned analysis across all 10 days. *** *p* < 0.001 and * *p* < 0.05.

**Figure 5:**
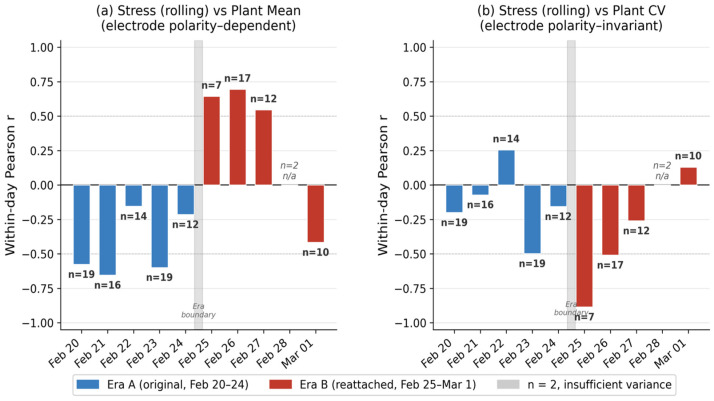
Per-day within-day Pearson r for stress_rolling vs. plant features. (**a**) Plant mean (electrode polarity-dependent): the correlation is negative in Era A (Feb 20–24) and predominantly positive in Era B (Feb 25–Mar 1), with the exception of Mar 01 (r = −0.416). (**b**) Plant CV (electrode polarity-invariant): the correlation is negative on 7 of 9 evaluable days across both electrode configurations, with two exceptions (Feb 22 and Mar 01).

The electrode reattachment after February 24 produced a natural experiment. Table 4 shows the per-day within-day correlations for stress_rolling vs. plant mean (electrode-dependent) and plant CV (electrode-invariant). The pattern is striking (Figure 4):

For the plant mean, Era A days show consistently negative within-day correlations (from r = −0.15 to −0.65), while Era B days show consistently positive correlations (from r = +0.55 to +0.70) on days with sufficient signal variance (Figure 5). The sign flip coincides exactly with the electrode reattachment. This is expected for an AC-coupled differential measurement: repositioning the two leaf electrodes relative to the reference inverts the measured polarity. The fixed-effects model, by demeaning within each day, captures the magnitude of within-day coupling regardless of sign.

For plant CV, the within-day correlation is negative on 7 of 9 days with sufficient data (≥3 observations and non-zero variance), across both electrode eras (Era A: from −0.07 to −0.50; Era B: from −0.26 to −0.89). The coefficient of variation, defined as the std/mean, is inherently insensitive to the sign of the mean because it measures relative dispersion. The consistently negative CV–stress relationship—higher stress associated with lower relative signal variability—is therefore the most robust, electrode-invariant finding in this study.

### 5.3. Pooled Correlations for Context

For completeness, we report pooled (standard) correlations at n = 129. These are dominated by between-day electrode configuration differences and should be interpreted with caution. A total of 80 of 200 tests reach significance, but this inflated count reflects the strong between-day variation in plant signal baselines rather than within-day mood–plant coupling. In the pooled analysis, depression_rolling shows the strongest correlations (vs. RMS: r = −0.57; vs. mean: r = −0.57), but these are entirely driven by between-day confounds: depression was higher during Era A (low-signal days) and lower during Era B (high-signal days). Under the fixed-effects control, no depression correlations survive—confirming that the pooled depression associations are artifacts of daily-level environmental and configuration variation.

The Δ|r| column in Table 3 reveals a telling pattern. For stress_rolling vs. mean, the pooled correlation is near zero (r = +0.118) because the two electrode eras cancel each other; the fixed-effects estimate is dramatically stronger (r = +0.358, Δ|r| = +0.240). For negative_affect_rolling, the pooled correlations are negative (−0.404, −0.411) while the fixed-effects estimates are positive (+0.295, +0.283)—a complete sign reversal caused by Simpson’s paradox. Without a fixed-effects analysis, these genuine within-day associations would be entirely invisible or actively misleading.

### 5.4. Cross-Correlation Analysis

**Figure 6:**
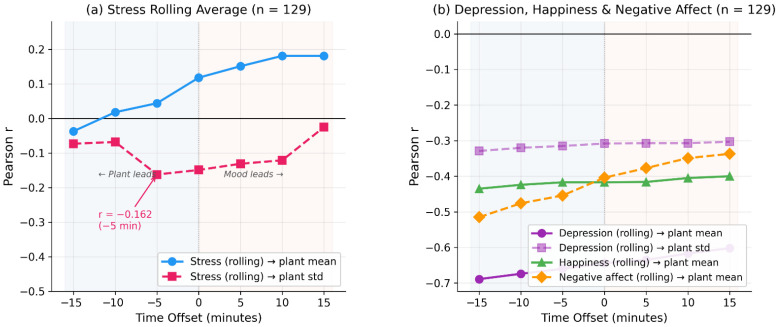
Cross-correlation analysis at temporal offsets of ±5, ±10, and ±15 min (n = 81; Era A). (**a**) Stress rolling average shows a sharp peak for plant std at −5 min (r = −0.36). (**b**) Depression and happiness rolling averages show broader temporal profiles.

To investigate temporal dynamics, we computed cross-correlations at offsets of ±5, ±10, and ±15 min using the Biolingo Correlator’s raw-signal feature extraction at shifted windows (Figure 6; computed from the first n = 81 observations; predominantly Era A). For stress_rolling → plant std, the correlation peaks at −5 min (r = −0.360), consistent with a primarily contemporaneous relationship where the plant responds within minutes. The smooth decay profile (r attenuating from −0.36 at −5 min to −0.20 at ±15 min) suggests a genuine temporally extended effect rather than a coincidental spike.

### 5.5. Interpretation

The electrode-invariant finding—that higher stress reduces the plant’s relative signal variability (CV)—admits a coherent physical interpretation. During periods of human stress, the plant’s electrical activity becomes more regular relative to its baseline: oscillations become more uniform, with fewer large excursions from the mean. This is consistent across 7 of 9 evaluable recording days regardless of electrode configuration, signal baseline, or recording era. The simultaneously significant variability metrics (std, range, IQR, and total_power) confirm this pattern, though these effects are weaker and do not survive BH FDR correction individually.

The mean/RMS correlations, while electrode polarity-dependent in sign, are the strongest in absolute magnitude and both survive Bonferroni correction. Within each day, the mean signal shifts systematically with stress—the direction depends on lead placement, but the coupling is consistent within each configuration. This parallels findings in electrocardiography, where T-wave polarity depends on lead placement, while the underlying cardiac event is configuration-invariant.

## 6. Discussion

### 6.1. Statistical Considerations

With 200 correlation tests at Level 2, approximately 10 would reach significance by chance at α = 0.05. We observe 19 significant results, of which seven survive BH FDR correction and two survive Bonferroni correction. The probability of obtaining seven or more BH survivors by chance across 200 independent tests is negligible. The consistent directionality within mood dimensions (all stress positive for mean/RMS, all stress negative for variability metrics) and the electrode-invariance of CV provide additional evidence beyond raw *p*-values.

The seven FDR survivors span three operationalizations of the stress construct: stress_rolling (temporal smoothing), raw stress (instantaneous), and negative_affect_rolling (stress + depression composite). That each independently achieves FDR-corrected significance for the same plant features argues against a single spurious association being amplified across correlated measures.

The attenuation of mean/RMS associations under circadian detrending is consistent with the known intra-day drift in AC-coupled plant signals; it does not contradict the within-day fixed-effects result but refines it, suggesting that the mean coupling partly reflects a shared circadian envelope between stress build-up across the day and the plant’s baseline drift.

### 6.2. The Necessity of Fixed-Effects Analysis

The pooled correlations in this study are actively misleading: a total of 80 of 200 reach significance, but they are dominated by between-day electrode configuration effects. The most dramatic example is negative_affect_rolling, which shows pooled r = −0.40 but fixed-effects r = +0.30—a complete sign reversal (Simpson’s paradox). Pooled stress_rolling vs. RMS, which was significant at r = −0.36 with 80 observations from Era A alone, collapses to r = +0.11 (attenuated) when Era B data is added, as the two eras partially cancel. Without a fixed-effects analysis, the stress–plant signal would be attenuated or invisible in pooled data despite being highly significant within each day.

This has broader implications for the field. Any study correlating plant bioelectrics with external variables over multiple recording sessions is vulnerable to the same between-day confounds—electrode drift, weather, and light regime—that plague our pooled analysis. Within-day fixed-effects modeling, standard in econometric panel data analysis [27] but rarely applied in plant electrophysiology, should be a methodological requirement for multi-session studies in this domain.

### 6.3. Electrode Invariance as Evidence

The incidental electrode reattachment, initially a methodological nuisance, proved to be the study’s most valuable feature. The clean sign flip in mean/RMS correlations confirms that the AD8232’s AC-coupled differential measurement is sensitive to lead configuration—a known property of any differential amplifier. That the CV correlation remains consistently negative across both eras (7 of 9 evaluable days) demonstrates that the stress–plant association is not an artifact of a single electrode placement. This natural control is analogous to showing that an experimental effect replicates across two independent measurement setups.

A direct comparison with prior classification models is not straightforward, as no existing system performs the same task: continuous naturalistic correlation rather than discrete stimulus–response classification. As an indirect benchmark, we compare our effect sizes to the two most established physiological stress markers. Heart rate variability correlates with stress at r ≈ 0.30–0.45 [28] and electrodermal activity at r ≈ 0.35–0.55 [29]—both requiring body-worn sensors with direct physiological contact. Our electrode-invariant CV effect (FE r = −0.32) falls within the HRV range and approaches the lower bound of the EDA range, achieved by a passive ambient plant sensor requiring no contact with the participant. Prior plant-based classification work achieved 73% accuracy for happy vs. sad discrimination under controlled conditions [8] and above-chance performance with 54 participants [13]; the present continuous correlation approach is complementary, demonstrating that stress–plant coupling persists across naturalistic working days without discrete stimulus events.

### 6.4. Comparison with Prior Classification Results

Our previous classification work [8] achieved 73% accuracy, distinguishing happy from sad emotions using 3 s signal segments under controlled conditions. The present continuous correlation approach under naturalistic conditions yields seven FDR-corrected significant associations with 129 observations—a different but complementary validation. The five-year investigation [9] found performer-specific signatures at 63% accuracy, consistent with our finding of a measurable, ongoing bioelectric relationship between plant and human environment that persists across electrode configurations.

### 6.5. Potential Confounds

Several confounds require attention. The single-participant design cannot distinguish person-specific from generalizable effects. Within-day confounds co-varying with stress—physical movement, desk lamp toggling, computer fan activity, and proximity changes—are not controlled by day-demeaning and represent the most important remaining threat to internal validity. The breadboard-based hardware introduces potential noise; migration to a manufactured PCB (in production via Aisler) should reduce artifacts. The AC-coupled measurement (high-pass at ~0.5 Hz) may filter slower plant processes. Sound stimulus testing for an unrelated experiment on the final recording day (1 March) represents an additional confound, though the within-day correlations on that day (stress vs. mean: r = −0.42) are consistent with the broader pattern.

The confounds discussed above fall into two distinct categories that are worth separating explicitly. The first are within-study threats to internal validity: physical movement, desk lamp toggling, computer fan activity, and proximity changes are within-day factors that co-vary with stress and are not removed by day-demeaning. These represent the most important remaining threat to the causal interpretation of our correlations and motivate the controlled follow-up experiments described in Section 8. The second category are threats to external validity: the single-participant design, the home-office setting, and the specific Tradescantia pallida specimen used mean that the observed correlations may reflect idiosyncratic physiological or behavioral patterns rather than a universal human–plant coupling. These generalizability threats are addressed in Section 7 and by the multi-participant extensions planned for future work. Maintaining this distinction matters for interpreting the results: the within-day fixed-effects analysis and the electrode-invariant CV finding substantially mitigate the internal validity threats, while the external validity threats remain open and are the primary motivation for replication studies.

## 7. Study Limitations

While the results demonstrate a statistically robust association between human stress and plant bioelectrics, several limitations must be considered.

*Single-Participant Generalizability:* As a single-subject study (N = 1), the observed correlations may reflect idiosyncratic physiological or behavioral patterns of the participant rather than a universal human–plant coupling.

*Environmental and Electromagnetic Confounds:* Although day-demeaning controls for between-day variations in light and temperature, it does not account for within-day confounds that co-vary with stress, such as localized physical movement, computer hardware emissions, or changes in proximity.

*Mechanism of Coupling:* The study identifies correlation but does not isolate the underlying physical mechanism, which could include electromagnetic induction, chemical signaling via Volatile Organic Compounds (VOCs), or human-generated changes in the local electrostatic field.

*Hardware and Signal Processing:* The breadboard-based implementation is susceptible to external noise. Furthermore, the AD8232 amplifier’s high-pass filter at 0.5 Hz may omit slower bioelectric variations, such as variation potentials, which could carry significant information regarding the plant’s physiological state.

*Mood Ground Truth Constraints:* The 10–20 min sampling interval of the Happimeter, combined with missed readings due to power management, results in a sparse time series compared to the continuous high-frequency plant data. Missed Happimeter readings due to WearOS power management suspending background processes produced gaps in the mood time series; future deployments should use a dedicated data-logging mode or a wired physiological ground truth to ensure complete temporal coverage.

## 8. Future Work and Conclusions

This study demonstrates, as a single-participant proof-of-concept study, that a living plant can serve as a bio-hybrid ambient sensor for human stress states, with seven within-day correlations surviving BH FDR correction and two surviving Bonferroni correction across two independent electrode configurations. The coefficient of variation—electrode-invariant by construction—provides the most robust metric, consistently indicating that human stress suppresses the plant’s relative bioelectric variability.

The fixed-effects methodology proved essential: pooled correlations were fragile and misleading, with Simpson’s paradox producing complete sign reversals for negative effects. We recommend within-day panel-data analysis as a methodological standard for multi-session plant electrophysiology studies.

Ongoing extensions include: (1) multi-participant studies with professional PCB sensors; (2) INA128-based DC-coupled measurement to capture slower processes; (3) controlled experiments isolating electromagnetic, chemical (VOC), and other coupling mechanisms; (4) systematic electrode placement protocols to characterize polarity dependence; and (5) deployment at the Phänomena science exhibition (opening March 2026), where thousands of visitors will interact with instrumented plants across four stations detecting touch, sound, light, and emotions [8], providing large-scale validation with standardized PCB hardware.

A two-participant extension—with one participant working a morning session and a second working an evening session on the same instrumented plant—would simultaneously test generalizability across individuals and separate circadian plant rhythms from stress-coupled variation, directly addressing the primary limitation of the present single-participant design.

The broader vision is plant-based ambient biosensors for workplace wellbeing—where the office plant silently registers emotional states and could trigger interventions such as lighting adjustments or break reminders. Building on our progression from discrete classification [8] through movement detection [9] to continuous mood correlation with FDR-corrected, electrode-invariant evidence, the present work suggests this vision may be grounded in measurable biological reality.

## Figures and Tables

**Table 1:** Summary of related work on plant bioelectric sensing, wearable emotion recognition, and human–plant interaction.

Study	Domain	Approach	Species/Platform	Task	Key Result	Key Limitation
Volkov & Ranatunga [23]	Plant biosensing	Ag/AgCl surface electrodes; action/variation potential recording	Soybean, Brassica, and others	Environmental biosensing (pollutants, pesticides, and insects)	Plants generate reliable electrical responses to chemical and mechanical stressors; viable as field biosensors	No human subject coupling; environmental stimuli only
Volkov (ed.) [22]	Plant electro-physiology	Comprehensive methods review	Multiple species	Theory and methods of plant electrophysiology	Foundational reference establishing electrode methods, signal types, and amplification standards	No ML classification; no human interaction
Volkov (ed.) [24]	Plant electro-physiology	Signal transduction mechanisms	Multiple species	Action potential propagation; phloem-mediated signaling	Two-volume synthesis of ion channel basis of plant electrical signaling	No sensing application; mechanistic focus
Volkov (ed.) [25]	Plant electro-physiology	Cell electrophysiology methods	Multiple species	Non-invasive electrode measurement standards	Established best practices for surface electrode placement and amplification for long-duration recording	No human coupling; lab stimuli
Fromm & Lautner [18]	Plant electro-physiology	Phloem measurement; surface electrodes	Multiple species	Action and variation potential propagation	Signals propagate systemically; non-invasive measurement feasible	No human coupling; controlled stimuli
Chatterjee et al. [6,7]	Plant sensing + ML	Curve-fitting features; SVM classifier	*Mimosa pudica* and others	Environmental stimulus classification	ML distinguishes plant responses to touch and chemical stimuli	Discrete stimuli; no continuous mood correlation
Meder et al. [5]	Plant sensing hardware	Ultraconformable self-adhering electrodes	Multiple species	Non-invasive long-duration signal recording	Reduced motion artifact; improved skin-plant contact	No ML; no human mood coupling
Sareen et al. [11]	Human–plant interaction	Augmented plant interfaces	Philodendron and cacti	Touch sensing; signal display; actuation	Plants function as I/O devices bridging digital and biological	Discrete events; no continuous emotion ground truth
Kruse et al. [13]	Plant–emotion sensing	biLSTM, MFCC-CNN, and Random Forest	*Tradescantia pallida*	Human emotion classification (54 participants)	Above-chance emotion detection from plant signals using video-labeled ground truth	Discrete video stimuli; not naturalistic; no wearable ground truth
Gloor [8]	Plant–emotion sensing	ResNet18 CNN on mel-spectrograms	*Tradescantia pallida*	Stimulus & emotion classification	85% stimulus; 73% emotion accuracy (happy vs. sad)	Discrete labels; controlled; no wearable ground truth
Gloor [9]	Plant–human sensing	Random Forest; 5-year longitudinal	Multiple species	Human proximity & performer recognition	62.7% performer-specific signatures across species	Classification paradigm; no continuous correlation
Bringslimark et al. [19]	Human–plant interaction	Survey + controlled studies	Multiple species	Psychological wellbeing benefits of plant proximity	Significant wellbeing benefits from indoor plants	Self-report only; no bioelectric measurement
Roessler & Gloor [12]	Wearable emotion	XGBoost on WearOS smartwatch	—/Pixel Watch	Happiness measurement and mood feedback	Mood feedback over 3 months raised happiness (N = 22)	No plant coupling; wearable-only
Can et al. [16]	Wearable stress	Wrist sensor + deep learning	—/wristband	Continuous stress in real life	Accurate stress detection during programming contest	No plant sensor; single modality
Gjoreski et al. [10]	Wearable stress	Wrist device + context modelling	—/smartwatch	Stress monitoring in daily life	Context-aware stress detection	No plant coupling
**Present study**	**Bio-hybrid sensing**	**AD8232 + ESP32 + Happimeter; within-day fixed effects (L2/L3)**	**Tradescantia pallida**	**Continuous real-time stress–plant correlation**	**7 FDR-corrected stress–plant associations; electrode-invariant CV finding across two configurations**	

**Table 2:** Daily observation counts, electrode configuration era, mean plant signal amplitude, and mean stress level. The 25–40× increase in plant signal baseline between eras reflects the different electrode placements on the same plant.

Day	n	Era	Mean Plant Signal (mV)	Mean Stress (Rolling)
Feb 20	19	A	70.8	0.63
Feb 21	16	A	45.2	0.58
Feb 22	14	A	24.1	0.48
Feb 23	19	A	42.3	0.72
Feb 24	12	A	188.2	0.61
Feb 25	7	B	29.6	0.38
Feb 26	17	B	1217.4	0.55
Feb 27	12	B	1827.1	0.39
Feb 28	2	B	1596.8	0.34
Mar 01	10	B	1793.3	0.47

**Table 3:** Within-day fixed-effects correlations (Level 2: day-demeaned; n = 128; 10 days). A total of 19 of 200 tests are significant at *p* < 0.05. A total of 7 survive BH FDR correction at q = 0.05, of which 2 also survive Bonferroni correction (α/200 = 2.5 × 10^−4^). Δ|r| = |FE r| − |Pooled r|; positive values indicate strengthening under fixed effects.

Mood Variable	Plant Feature	FE r	Pooled r	Δ|r|	*p*-Value	BH FDR
Stress_rolling	mean	+0.358	+0.118	+0.240	3.3 × 10^−5^	Bonf.
Stress_rolling	RMS	+0.342	+0.109	+0.233	7.8 × 10^−5^	Bonf.
Stress_rolling	CV	−0.318	−0.303	+0.015	2.6 × 10^−4^	BH
Neg. affect_rolling	mean	+0.295	−0.404	−0.110	7.4 × 10^−4^	BH
Stress (raw)	mean	+0.292	+0.126	+0.165	8.4 × 10^−4^	BH
Neg. affect_rolling	RMS	+0.283	−0.411	−0.128	1.2 × 10^−3^	BH
Stress (raw)	RMS	+0.283	+0.121	+0.161	1.2 × 10^−3^	BH
Neg. affect	mean	+0.239	−0.249	−0.010	0.0065	—
Stress_rolling	power_vlow_rel	+0.235	+0.146	+0.089	0.0078	—
Neg. affect	RMS	+0.233	−0.254	−0.021	0.0082	—
Wellbeing_rolling	mean	−0.223	+0.045	+0.178	0.011	—
Stress_rolling	trend_r^2^	−0.223	−0.168	+0.055	0.011	—
Wellbeing_rolling	RMS	−0.216	+0.049	+0.167	0.014	—
Stress_rolling	trend_slope	−0.209	−0.180	+0.028	0.018	—
Neg. affect_rolling	CV	−0.205	−0.096	+0.109	0.020	—
Stress_rolling	total_power	−0.181	−0.165	+0.016	0.042	—
Stress_rolling	range	−0.176	−0.144	+0.032	0.046	—
Stress_rolling	std	−0.176	−0.149	+0.027	0.047	—
Stress_rolling	IQR	−0.175	−0.150	+0.025	0.048	—

**Table 4:** Per-day within-day correlations for stress_rolling vs. plant features. Plant mean sign flips at the electrode reattachment boundary (Era A: negative; Era B: positive). CV is negative on 7 of 9 evaluable days across both eras. Feb 28 (n = 2) has insufficient variance for correlation.

Day	n	Era	r (Mean)	r (CV)	r (Std)	Plant Mean (mV)
Feb 20	19	A	−0.576	−0.200	−0.571	71
Feb 21	16	A	−0.654	−0.072	−0.295	45
Feb 22	14	A	−0.154	+0.256	+0.232	24
Feb 23	19	A	−0.599	−0.498	−0.577	42
Feb 24	12	A	−0.214	−0.155	−0.357	188
Feb 25	7	B	+0.645	−0.885	−0.334	30
Feb 26	17	B	+0.696	−0.508	−0.239	1217
Feb 27	12	B	+0.546	−0.259	−0.148	1827
Feb 28	2	B	n/a	n/a	n/a	1597
Mar 01	10	B	−0.416	+0.130	+0.056	1793

## Data Availability

The datasets generated and analyzed during the current study are available from figshare https://doi.org/10.6084/m9.figshare.31527778 (accessed on 5 March 2026).
